# Editorial: Electrocatalysis on Shape-Controlled Nanoparticles

**DOI:** 10.3389/fchem.2019.00885

**Published:** 2019-12-20

**Authors:** Paramaconi Rodriguez, José Solla-Gullón

**Affiliations:** ^1^School of Chemistry, University of Birmingham, Birmingham, United Kingdom; ^2^Institute of Electrochemistry, University of Alicante, Alicante, Spain

**Keywords:** electrochemistry, electrocatalysis, shape control, nanoparticles, surface structure sensitivity

The incorporation of shape-controlled metal nanoparticles in electrocatalysis is improving the activity, selectivity, and stability of many different electrocatalytic reactions of interest (Hong et al., 2016; Strasser et al., 2018; García-Cruz et al., 2019; Rizo and Roldan Cuenya, 2019). It is well-established, by simple crystallographic analogies, that the shape of a nanoparticle strongly determines the coordination of the atoms at its surface; that is, its surface structure (Zhou et al., 2011). Consequently, the application of such shaped nanoparticles is also deeply contributing to a better understanding of the effect of the surface structure at nanoscale for many electrocatalytic processes. However, despite significant contributions, much more work is still required to fully visualize the capabilities of these nanomaterials (Pan et al., 2019).

Among the factors that must be taken into consideration, the surface cleanliness is one of the most critical. Several synthetic approaches can produce shaped nanoparticles, but the use of surface capping agents under colloidal routes is one of the most effective ways to control the growth of nanoparticles so as to induce the formation of specific particle shapes (Xia et al., 2015; Yang et al., 2019). However, once nanoparticles are formed, the presence of these surface-regulating agents at the surface of the nanoparticles results in an obstacle; and it is mandatory to apply specific decontamination protocols to completely remove (without altering the initial surface structure of the nanoparticles) their presence from the surface of the nanoparticles (Monzó et al., 2012; Montiel et al., 2017; García-Cruz et al., 2019). This is a critical step that has been generally underestimated in literature dealing with the electrocatalytic properties of shaped nanoparticles. Devivaraprasad et al. reported a comprehensive review dealing with some of the most important aspects of the use of shaped Pt and Pd nanoparticles including, (i) methods of synthesis of these nanoparticles, (ii) decontamination procedures to effectively remove the adsorbed capping agents and/or surfactants without disturbing the surface structure, (iii) electrochemical reactivity of these shaped nanoparticles toward the oxygen reduction reaction (ORR), and (iv) stability of the nanoparticles under electrochemical conditions. This review presents an ideal overview of all aspects which must be taken into consideration to properly employ these nanoparticles for any electrocatalytic study.

Getting detailed and statistically representative information about the surface structure of the shaped nanoparticles is also of fundamental importance because the key point controlling the electrocatalytic activity is the surface structure of the nanoparticles (obviously, the atomic composition is also extremely relevant), not the particle shape (Vidal-Iglesias et al., 2011). In this regard, the use of electrochemical tools has allowed a detailed characterization of the surface structure of different shaped Pt and Au nanoparticles (Rodríguez et al., 2005; Solla-Gullón et al., 2008; Monzo et al., 2015; Jeyabharathi et al., 2018). Garnier et al. discussed the use of the Cu UPD to characterize the surface structure of different shape controlled Pd nanoparticles. The results obtained indicate that Cu UPD allows, based on Pd single crystal features, a qualitative and quantitative analysis of the surface structure of different shape-and-size-controlled Pd nanoparticles to be performed. This detailed characterization is of fundamental interest to properly establishing and understanding the correlations between electrocatalytic activity and surface structure at nanoscale.

The modification of the surface of the shaped nanoparticles with some adatoms is also an interesting approach to enhance the activity, stability, and selectivity of different electrocatalytic reactions of interest (de Souza et al., 2018; Dionigi et al., 2019). Rafaïdeen et al. studied the electrooxidation of glucose in alkaline medium on bare and Au-modified shape-controlled Pd nanoparticles. On non-modified nanoparticles, those with a higher density of {100} domains displayed higher activity. For the Au-modified samples, and independent of the particle shape, the resulting activity is much higher than that obtained with bare samples; although, the activity is clearly dependant on the Au coverage. However, the Au-modified Pd nanocubes still exhibited the highest activity toward glucose electrooxidation. Under the same concept, Hong et al. reported the preparation of Pt-Cu octahedral nanoparticles decorated with Ni(OH)_2_. The authors also evaluated the catalytic activity of the nanocatalyst toward the electrochemical oxidation of ethanol and carbon monoxide in acidic solution. It is shown that the Ni(OH)_2_-decorated samples displayed enhanced CO tolerance relative to the bare nanoparticles, due to the presence of Ni(OH)_2_ at their surface. Even more, both Ni(OH)_2_-decorated and bare Pt-Cu octahedral samples show enhanced activity and durability during ethanol electrooxidation, in comparison with commercial Pt/C electrocatalysts.

Developing non-platinum-group-metal (PGM)-based electrocatalysts is also of great interest; in particular, as catalysts for the ORR in Polymer Electrolyte Membrane Fuel Cells (Rabis et al., 2012). Khan et al. reported the use of a novel, two-dimensional, mesoporous CrN-reduced graphene oxide (rGO) nanocomposite with MnO as an electrocatalyst toward the ORR in alkaline solution. This non-PGM-based electrocatalyst displays enhanced ORR properties relative to a benchmark 20% wt Pt/C electroacatalyst. In addition, the nanocomposite also shows an interesting methanol tolerance and improved stability in alkaline media. Preliminary fuel cell testing [alkaline anion exchange membrane fuel cell (AAEMFC)] provided a maximum power density of about 309 mW cm^−2^, surpassing the maximum power of 250 mW cm^−2^, obtained with the benchmark 20% wt Pt/C electroacatalyst.

Theoretical calculations are strongly impacting and guiding the development of new electrocatalysts (Greeley et al., 2006; Nørskov et al., 2009). Lozano and Rankin reported relevant DFT calculations, studying the effect of particle size and the incorporation of some dopants in different graphene-nanoparticle systems. The adsorption energy of mono-atomic oxygen O* was used as descriptor of the ORR activity. Several sub-nanometer (4, 7, and 19-atoms) binary alloy/intermetallic transition metal nanoparticle (Ni-Cu, Au-Pd, Rh-Ir, Cu-Ni, and Pt-Pd) catalysts on different doped graphene were studied. The results obtained with doped-graphene-Ni_x_Cu_y_ systems were found to provide the necessary shifts in O* binding energies to reach the predicted activity maximum in the volcano plot for the ORR.

As Guest Editors of this Research Topic, we would like to thank all the authors and reviewers for their valuable contributions to this special issue. We expect that the readers find this topic issue of interest.

## Author Contributions

All authors listed have made a substantial, direct and intellectual contribution to the work, and approved it for publication.

### Conflict of Interest

The authors declare that the research was conducted in the absence of any commercial or financial relationships that could be construed as a potential conflict of interest.
